# Minireactor-based high-throughput temperature profiling for the optimization of microbial and enzymatic processes

**DOI:** 10.1186/1754-1611-8-22

**Published:** 2014-08-04

**Authors:** Martin Kunze, Clemens Lattermann, Sylvia Diederichs, Wolfgang Kroutil, Jochen Büchs

**Affiliations:** 1 AVT-Chair for Biochemical Engineering, RWTH Aachen University, Worringerweg 1, 52074 Aachen, Germany; 2 Department of Chemistry (Organic and Bioorganic Chemistry), University of Graz, Heinrichstraße 28/II, 8010 Graz, Austria

**Keywords:** High-throughput screening, On-line monitoring, Microtiter plate, Optical measurement, Temperature optimum

## Abstract

**Background:**

Bioprocesses depend on a number of different operating parameters and temperature is one of the most important ones. Unfortunately, systems for rapid determination of temperature dependent reaction kinetics are rare. Obviously, there is a need for a high-throughput screening procedure of temperature dependent process behavior. Even though, well equipped micro-bioreactors are a promising approach sufficient temperature control is quite challenging and rather complex.

**Results:**

In this work a unique system is presented combining an optical on-line monitoring device with a customized temperature control unit for 96 well microtiter plates. By exposing microtiter plates to specific temperature profiles, high-throughput temperature optimization for microbial and enzymatic systems in a micro-scale of 200 μL is realized. For single well resolved temperature measurement fluorescence thermometry was used, combining the fluorescent dyes Rhodamin B and Rhodamin 110. The real time monitoring of the microbial and enzymatic reactions provides extensive data output. To evaluate this novel system the temperature optima for *Escherichia coli* and *Kluyveromyces lactis* regarding growth and recombinant protein production were determined. Furthermore, the commercial cellulase mixture Celluclast as a representative for enzymes was investigated applying a fluorescent activity assay.

**Conclusion:**

Microtiter plate-based high-throughput temperature profiling is a convenient tool for characterizing temperature dependent reaction processes. It allows the evaluation of numerous conditions, e.g. microorganisms, enzymes, media, and others, in a short time. The simple temperature control combined with a commercial on-line monitoring device makes it a user friendly system.

## Background

Bioprocesses, either fermentations or enzymatic catalysis, depend on a number of different operating parameters. Temperature is one of the most important. It is commonly known that processes should be performed under optimal conditions in order to achieve best results, e.g. reaction rates or yields [1]. Thereby, the temperature optimum can be different within one microbial or enzymatic system, either the focus is on growth or product formation [2], on enzyme activity or stability [3,4].

Over the last decades the number of potential bioprocesses for the production of valuable products increased and this trend continues. Consequently, more and more microbial and enzymatic systems need to be characterized regarding their temperature optima. Conventional methods are disadvantageous in many ways. Repetitive batch cultivations at varied temperatures, either in shake flasks or bench scale bioreactors, provide only a few data points at the expense of relatively high material and time input. The analysis of temperature-specific activities and kinetic parameters of enzymes is traditionally performed in a spectrophotometric way by use of a temperature-controlled water jacketed single cuvettes [5]. Even though the external thermostat provides constant and accurate temperature control over a broad temperature range, the numbers of samples handled for simultaneous reading in such spectrophotometers are usually very limited.

To face these limitations micro-bioreactors (MBR) became a promising alternative, but sufficient temperature control is quite challenging [6]. The simplest way is to use MBRs, e.g. microtiter plates (MTP), in temperature controlled rooms [7] or incubators [8]. Another option is to link the MBR system to a thermostat system and circulate water through the MBR chamber base [4,9-11]. The major disadvantage of these systems is their limitation in operating parallel reactors at different temperatures. One exceptional system allows the operation of sixteen parallel small scale reactors equipped with ceramic heating jackets but its use requires costly instrumentation and is focused on chemical reactions [12]. The integration of electrical micro-heaters to the bottoms or walls of MBRs allows individual temperature control of parallel MBRs [13-15]. However, for high-throughput application the required hardware and control become exceedingly complex.

Whilst temperature control is rather challenging in MBRs, temperature measurement is relatively simple. There are several reports on the application of temperature dependent resistances [14-16] or thermocouples [10,17]. Also thermal or infrared cameras allow temperature determination in MBRs [18,19]. Furthermore, the so called fluorescence thermometry can be used [11,20-22]. The combination of a temperature dependent fluorophore and a spectrophotometer is a promising method for high-throughput temperature determination, e.g. in MTPs.

In this work, a system is presented combining the on-line monitoring system BioLector [23,24] with a customized temperature control unit for 96 well MTPs, thereby, allowing high-throughput temperature optimization for microbial and enzymatic reaction systems in a micro-scale of 200 μL. On the one hand, the BioLector system allows quasi-continuous measurement of optical signals over time representative for microbial growth and product formation or enzymatic activity. On the other hand, the tempering system, consisting of a special thermostating block connected to two thermostats, creates specific temperature profiles over a MTP. For the single well temperature measurement fluorescence thermometry was used. Combining the fluorescent dyes Rhodamin B (RhB) and Rhodamin 110 (Rh110) a reliable assay was established for application with the BioLector technique [21]. After defining several profiles at different temperature levels, the device was used to characterize microbial and enzymatic systems. The whole temperature profiles and optima for the bacterium *Escherichia coli* and the yeast *Kluyveromyces lactis* regarding growth and recombinant protein production were determined in single shot experiments. Additionally, the commercial cellulase mixture Celluclast as a representative for enzymes was investigated applying a fluorescent activity assay. The results of this temperature high-throughput screening could be used for a mathematical description of the particular temperature dependent behavior of the investigated biological system as an extended Arrhenius model for catalyst activation and deactivation [25].

## Material and methods

### Temperature control unit

**Figure 1:**
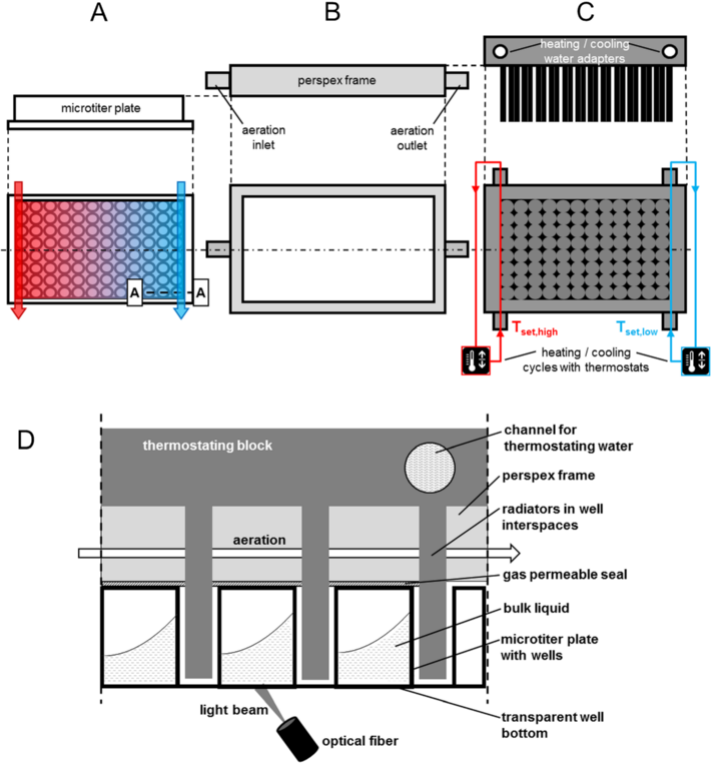
**Scheme of the temperature control unit for temperature profiling in microtiter plates (MTP). (A)** Side view on the MTP and top view with schematic illustration of thermostating water flows (arrows) and resulting temperature profile. **(B)** Side and top view on the perspex frame for aeration **(C)** Side view on the thermostating block and bottom view with schematic illustration of the heating (red) and cooling (blue) water circulation and their two respective thermostats. T_set,high_ and T_set,low_ stand for thermostat set point temperatures for heating and cooling water, respectively. **(D)** Cut A-A-view (see Figure 1A) on the microtiter plate with attached thermostating block and optical measurement.

The design of the heating block is based on the work of Rachinskiy et al. [4,9]. It is made of aluminium with tube adapters for the two water circulation systems (Figure 1C). The circulation system for cooling was operated with the FL300 recirculation cooler (Julabo, Seelbach, Germany) allowing temperature set points from −20°C to 40°C. The flow rate was determined to 2.1 L min^−1^. The heating water circulation worked with the Ecoline E300 thermostat (Lauda, Lauda-Königshofen, Germany) for temperature set points of 25-100°C. A flow rate of 1.1 L min^−1^ was measured. Both circulation systems were operated with tap water. Temperature set points below 5°C and above 95°C were avoided to prevent the water from freezing or boiling.

### On-line monitoring system

The optical measurement technique is identical to that described in the work of Samorski et al. [24]. The in-house constructed device is henceforth referred as BioLector. Wavelengths and gain factors for all optical signals are specified in Table 1. For all experiments 96well lumox multiwell plates (Greiner Bio-One GmbH, Frickenhausen, Germany) were used.

**Table 1:** Optical signals and applied setup for on-line monitoring

**Optical signal**	**λ**_**ex **_**[nm]**	**λ**_**em **_**[nm]**	**Gain**
Rhodamin B fluorescence	485	589	20
Rhodamin 110 fluorescence	485	520	2
Biomass (scattered light)	620	-	20
FbFP fluorescence	460	490	45
GFP fluorescence	485	510	30
4-methylumbellyferone fluorescence	365	455	25

### Temperature determination

For the temperature measurement via fluorescent dyes a mixture of Rhodamin B (RhB) and Rhodamin 110 (Rh110) was used. Before each experiment a fresh measuring solution was prepared from 100-fold concentrated stock solutions of RhB and Rh110, respectively. For these stock solutions dye powder was dissolved in pure methanol yielding a concentration of 1 g L^−1^ and stored at 4°C. For the measuring solution the two stock solutions were mixed with water and diluted to a final concentration of 10 mg L^−1^, respectively. 200 μL of the prepared solution were filled into each well of the MTP and shaken at a frequency of 995 rpm at a shaking diameter of 3 mm.

For temperature reference a special in-house constructed PT100 temperature sensor was placed in the arbitrary chosen well A2 of the microtiter plate. The sensor was connected to the serial port of the LAUDA thermostat for data output. Before use the sensor was calibrated with a gauged thermometer.

For the calibration of the fluorescence signal versus the temperature only the well equipped with the PT100 sensor was monitored in order to accelerate the procedure. For each point five consecutive measuring values were averaged. For temperature profiles twelve consecutive measurement cycles of the whole MTP were performed to get the average for each well. The PT100 sensor was placed as reference in well A2. For each specific temperature profile fresh prepared measuring solution with a new determined calibration curve was used.

### Microorganisms

The *E. coli* clone expressing FbFP was *E. coli* BL21 (DE3) with the pRhotHi vector and kanamycin resistance. For alcohol dehydrogenase A (ADH-A, from *Rhodococcus ruber*) expression *E. coli* BL21 (DE3) with the pET22b vector and ampicillin resistance was used. For *K. lactis* experiments the strain GG799 with the pKlac1 vector expressing GFP was applied.

### Media and cultivation

For *E. coli* pre-cultures terrific broth (TB) medium consisting of 12 g L^−1^ tryptone, 24 g L^−1^ yeast extract, 12.54 g L^−1^ K_2_HPO_4_, 2.31 g L^−1^ KH_2_PO_4_, and 5 g L^−1^ glycerol (all ingredients from Roth, Germany) dissolved in water was used. The pH value was 7.2 ± 0.2 without adjustment. For the main cultivation of *E. coli* under non-induced conditions either TB medium or a modified Wilms and Reuss medium (henceforth referred as Wilms-MOPS medium) were used [26,27]. Wilms-MOPS medium consists of 5 g L^−1^ (NH_4_)_2_SO_4_, 0.5 g L^−1^ NH_4_Cl, 3.0 g L^−1^ K_2_HPO_4_, 2 g L^−1^ Na_2_SO_4_, 0.5 g L^−1^ MgSO_4_ · 7H_2_O, 0.01 g L^−1^ thiamine hydrochloride, 20.9 g L^−1^ 3-(N-morpholino)-propanesulfonic acid (MOPS, 0.2 M), 20 g L^−1^ glucose and 1 mL L^−1^ trace element solution. This trace element solution consists of 1.98 g L^−1^ CaCl_2_ · 2H_2_O, 0.54 g L^−1^ CoCl_2_ · 6H_2_O, 0.48 g L^−1^ CuSO_4_ · 5H_2_O, 41.76 g L^−1^ FeCl_3_ · 6H_2_O, 0.3 g L^−1^ MnSO_4_ · H_2_O, 0.54 g L^−1^ ZnSO_4_ · 7H_2_O, 33.39 g L^−1^ Na_2_EDTA (Titriplex III). The pH was adjusted with 5 M NaOH to a value of 7. For experiments under induced conditions either the commercially available ready-made complex auto-induction medium Overnight Express Instant TB medium (OnEx, Novagen®/Merck, Darmstadt, Germany) or a modified Wilms-MOPS medium was used. OnEx consists of complex components similar to TB medium and the carbon sources glucose, lactose, and glycerol. For preparing the medium 60 g of the commercial granulate and 12.6 g of glycerol were dissolved in water and filled up to 1 L without pH-adjustment. HPLC analysis of the medium indicated a glucose concentration of 0.5 g/L and a lactose concentration of 2 g/L. For the modification of Wilms-MOPS medium in order to get a mineral auto-induction medium 1 g L^−1^ glucose, 4 g L^−1^ lactose and 5 g L^−1^ glycerol where added instead of 20 g L^−1^ glucose as carbon source. Depending on the clone’s resistance, 50 μg mL^−1^ kanamycin or 100 μg mL^−1^ ampicillin were added to the medium from a 1000 fold concentrated stock solution.

For *K. lactis* pre-cultures and cultivation under non-induced conditions yeast extract peptone (YP) [28] medium was used, consisting of 10 g L^−1^ yeast extract, 20 g L^−1^ tryptone and 20 g L^−1^ glucose. For cultivations under induced conditions 20 g L^−1^ galactose instead of glucose served as carbon source and inducer for recombinant protein expression.

For *E. coli* pre-cultivation, 10 mL of TB medium in a 250 ml shake flask were inoculated with 50 μL from a cryoculture, and cultures were grown for 8 h at 350 rpm (shaking diameter 50 mm) and 37°C. *K. lactis* pre-culture conditions were the same aside from being grown in YP medium for 12 h at 30°C.

For all main cultivations the respective medium was inoculated from the pre-culture, resulting in an initial OD_600_ of 0.1. 200 μL of the already inoculated medium was then transferred to each of the wells of the MTP. The plates were sealed with gas-permeable seals (AB-0718, Thermo Scientific, Dreieich, Germany). Subsequently, the pre-tempered thermostating block was mounted atop the MTP and both were fixed on the orbital shaker (Kühner AG, Basel, Switzerland) of the BioLector. The cultivation was performed at a shaking frequency of 995 rpm and a shaking diameter of 3 mm. For aeration pure oxygen was used. For scattered light and fluorescence measurement the initial light intensity (I_0_), which is mainly attributed to such factors as the media background or the type of the microtiter plate, was subtracted from the original measured data (I-I_0_).

Reference shake flask cultures were performed in a Respiration Activity Monitoring System (RAMOS) [29,30] built in-house. Commercial versions of this device are available from Hitec Zang (Herzogenrath, Germany) or Kühner AG (Birsfelden, Switzerland). 10 mL of OnEx medium in a special 250 ml shake flask were inoculated from the pre-culture, resulting in an initial OD_600_ of 0.1. Subsequently, the cultures were shaken at 350 rpm with a shaking diameter of 50 mm at temperatures of 22-37°C with aeration by pressurized air. Cells for ADH-A analysis were harvested in the stationary phase, indicated by the OTR on-line signal of the RAMOS device.

### Cellulase experiments

For hydrolysis experiments the substrate 4-methylumbelliferyl-β-D-cellobioside (4MUC) was used in combination with the commercial cellulase mix Celluclast 1.5 L (Novozymes, Bagsvaerd, Denmark). A 0.75 mM stock solution of 4MUC in 0.1 M acetate buffer (pH = 4.8) was prepared. The enzyme stock solution contained 5 g L^−1^ of Celluclast crude extract in 0.1 M acetate buffer (pH = 4.8). Before starting the experiments, 180 μL of the 4MUC stock solution were filled in each well of a 96 well MTP and pre-heated for 30 min applying the respective temperature profile. Subsequently, 20 μL of the enzyme stock solution were added to each MTP well resulting in final concentrations of 0.6 mM 4MUC and 1 g L^−1^ Celluclast. Since no aeration was necessary, the plate was sealed with a non-permeable foil (AB-0580, Thermo Scientific, Dreieich, Germany). The further procedure was identical to the cultivation experiments in MTPs besides that 4-methylumbellyferone (4MU) fluorescence was the only measuring signal (Table 1). To calibrate the fluorescence signal, 200 μL of solutions with varied 4MU concentrations were filled in a 96 well MTP and their fluorescence intensity was measured using the BioLector. The calibration measuring points are average values from three independent measurements.

### Offline analysis

OD_600_ was determined via a Genesys 20 photometer (Thermo Scientific, Dreieich, Germany) in 1.5 mL micro cuvettes (PS, Plastibrand, Roth, Karlsruhe, Germany). For values higher than 0.5 the samples were appropriately diluted with 0.9% [wt/vol] NaCl solution.

The volumetric activity of the produced alcohol dehydrogenase A (ADH-A) was determined at 30°C by following the oxidation of NADH at a wavelength of 340 nm in 96-well microtiter plates (F-profile, Roth, Germany) using a Synergy-4 Multi-Mode Microplate Reader (BioTek Instruments, Germany). For cell disruption of *E. coli* expressing ADH-A, the cell pellet of 500 μL culture broth was suspended in 100 μL BugBuster Protein Extraction Reagent (Novagen, Merck, Germany) adding 1000 U/mL lysozyme (Roth, Germany) and 25 U/mL DNaseI (AppliChem, Germany). Cell disruption was continued according to the manufacturers’ specifications obtaining the soluble fraction with dissolved ADH-A. 200 μL reaction mixture (including enzyme solution) were prepared for measurement of ADH-A activity and contained 50 mM Tris buffer (pH 8, RT), 100 mM 2,5-hexanedione, and 0.5 mM NADH (biomol, Germany). By addition of the enzyme solution the reactions were initiated, thereby, applying appropriate enzyme solution amounts to ensure linear decreases of NADH absorbance over 1 min at least. One unit (U) was defined as the amount of enzyme converting 1 μmol cofactor per min.

## Results and discussion

### Development of the instrumentation

The here used measurement setup is a modification of an earlier developed system, the so called Enzyme Test Bench [4,9]. It combines an optical on-line monitoring system for cultivations and reactions in microtiter plates with a special system for temperature regulation.

The temperature control unit is depicted in Figure 1. It is similar to the heat transfer block of the Enzyme Test Bench for oxygen consuming reactions described by Rachinskiy et al. [9]. During experiments the MTP (side and top views in Figure 1A) is covered by a perspex frame (side and top views in Figure 1B) with an inlet and an outlet for aeration. The thermostating block (side and top views in Figure 1C) is mounted on top. It includes radiators which fit exactly within the well interspaces. Thus, the wells are jacketed by these radiators providing efficient heat transfer between the thermostating block and the MTP wells. The heat transfer properties of the system were specified before [4]. The whole system itself was placed in a temperature controlled environment. To minimize the evaporation, a gas permeable seal is fixed between the MTP and the perspex frame. In this foil a pattern in the radiators’ shape is cut. Additionally, the aeration gas is passed through five bubble columns to saturate it with water vapor. A sectional view (A-A-cut in Figure 1A) of the MTP attached with the perspex frame and thermostating block is shown in Figure 1D.

The original thermostating block described by Rachinskiy et al. was designed to ensure a homogeneous temperature distribution over the microtiter plate [9]. Contrary to that, in this work the aim was to create a temperature gradient. For this purpose, the thermostating block has two water circulation systems instead of one, each with a separate thermostat (Figure 1C). If operated at different temperatures a certain temperature profile can be imposed onto the MTP just by heat conductance through the aluminium block (Figure 1A).

The advantage of the thermostating block mounted on top of the MTP, compared to systems working e.g. with electrical micro-heaters (9, 23, 34, 36) is that the transparent MTP bottom is still available for optical measurements. The applied on-line monitoring system (indicated by the optical fiber and light beam in Figure 1D) is similar to the BioLector technique presented before [24]. Thereby, the equipped fluorescence spectrometer allows measurements at different wavelengths. With this setup, microbial growth (via scattered light) or the formation of various fluorescent components is continuously followed in microtiter plates without interruption of the shaking process.

### Optical temperature determination in MTPs

**Figure 2:**
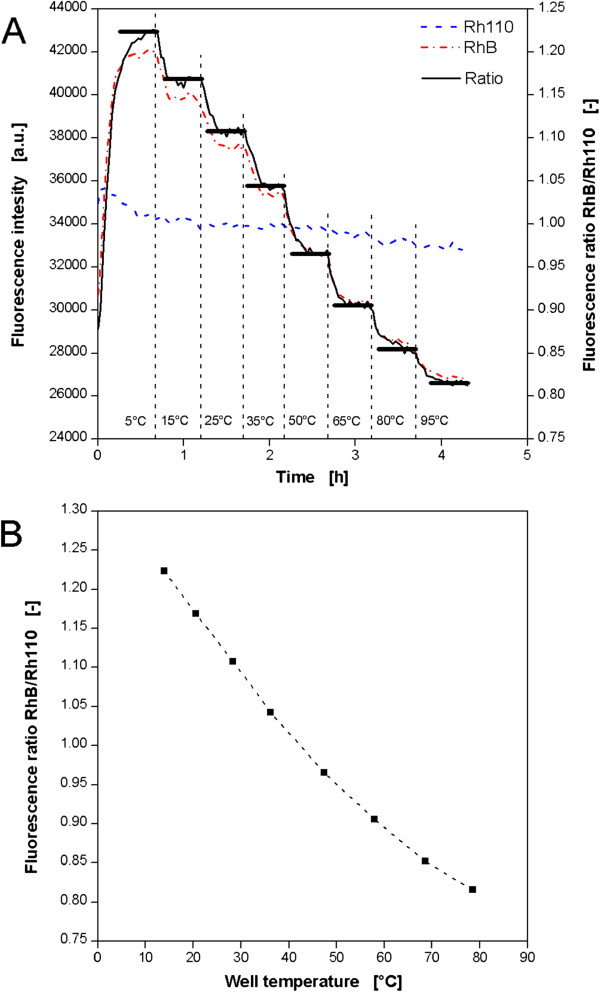
**Optical temperature measurement in MTPs applying a fluorescent assay with the dyes Rhodamin B and Rhodamin 110. (A)** Progress of the fluorescence signals of Rhodamin B (RhB) and Rhodamin 110 (Rh110) and their calculated ratio in a single well with varied thermostat set point temperature. Contrary to Figure 1C heating and cooling circulation systems were operated with one thermostat. **(B)** Calibration curve of the well temperature (measured via PT100 thermometer) vs. the fluorescence ratio RhB/Rh110. Experimental conditions: 96well MTP, V_L_ = 200 μL, n = 995 rpm, d_0_ = 3 mm, RT = 37°C.

To characterize the behavior of microbial or enzymatic systems at different temperatures it is necessary to know the specific temperature in each single well. Equipping each well with a temperature sensor requires a very high degree of instrumentation. Just by using a temperature dependent fluorophore measurements can easily be done with the optical on-line monitoring system. In this work a combination of the fluorescent dyes Rhodamin B (RhB) and Rhodamin 110 (Rh110) was applied, where RhB is the temperature sensitive compound, whereas Rh110 acts as a reference. This measuring principle was described before [21]. In Figure 2A the fluorescence intensity of both dyes depending on the temperature is shown. Therefore, the thermostating block was tempered only by one thermostat to ensure a constant temperature distribution over the whole MTP. Thermostat set point temperatures from 5-95°C were adjusted. After each temperature shift (dotted vertical lines) the experimental conditions remained unchanged until both fluorescence signals showed constant values. The RhB signal decreases in a step like manner since its fluorescence intensity is decreasing with increasing temperature. After each temperature shift the fluorescence signal drops sharply before it remains constant when the temperature reaches its equilibrium. On the contrary, the fluorescence intensity of Rh110 is almost constant over the whole time. The slight decrease is due to a bleaching effect which is known to happen to RhB as well. To obtain a reliable measuring signal, the ratio of both fluorescence intensities was calculated [21].It must be considered that the thermostat set point temperatures in Figure 2A and the actual temperatures in the MTP’s wells are not identical since heat may be lost to the environment. For this reason, one well was equipped with an in-house constructed PT100 temperature sensor. In this way, the corresponding well temperatures for various RhB/Rh110 ratios were determined. In Figure 2B the resulting calibration curve is depicted. It is described by a polynomial equation of second degree applying MS Excel. The fluorescence ratios are average values of five measurements in one well. The maximum relative standard deviation was 0.4%. For further investigation of the measuring accuracy the heating block temperature, as well as the room temperature, was adjusted to 37°C. In this way, a constant temperature of 37°C in each well could be assumed. The regarding measurement of all 96 wells revealed an average value of 37°C with a maximum of 38.7°C and a minimum of 35.6°C. The standard deviation was 0.76 K. The reason for the deviation from well to well cannot be explained completely. Slight deviations in the properties of the transparent microtiter plate bottom are possible which may influence the optical signals. A systematic position effect could be excluded during the experiments.

### Temperature profiles

**Figure 3:**
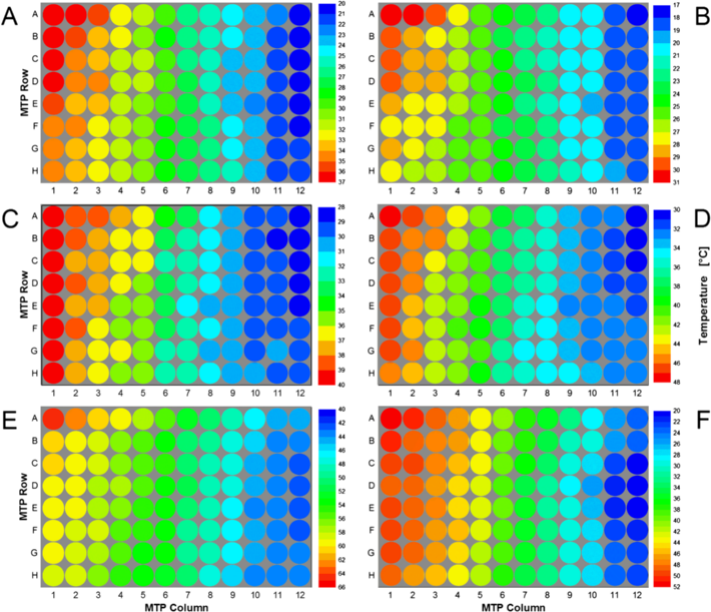
**Exemplary temperature profiles in MTPs at varied room temperature and thermostat set point temperatures. (A)** RT = 30°C, T_set,low_ = 5°C, T_set,high_ = 50°C, n = 995 rpm. **(B)** RT = 30°C, T_set,low_ = 5°C, T_set,high_ = 40°C, n = 995 rpm. **(C)** RT = 37°C, T_set,low_ = 5°C, T_set,high_ = 50°C, n = 995 rpm. **(D)** RT = 37°C, T_set,low_ = 5°C, T_set,high_ = 60°C, n = 995 rpm. **(E)** RT = 37°C, T_set,low_ = 10°C, T_set,high_ = 95°C, n = 995 rpm. **(F)** RT = 37°C, T_set,low_ = 5°C, T_set,high_ = 60°C, n = 0 rpm. Note: Temperature scale differs from A to F for better visualization of temperature profiles. Experimental conditions: 96well MTP, V_L_ = 200 μL, d_0_ = 3 mm.

The optical temperature measuring method was then used to characterize the temperature distribution over microtiter plates at varied set point temperatures of the heating and cooling thermostat (T_set,high_, T_set,low_) and at varied room temperature (RT). In Figure 3A the exemplary temperature profile for T_set,low_ of 5°C, T_set,high_ of 50°C and a RT of 30°C is depicted. As expected, the profile shows a clear gradient from the warm right side (column 1) to the cold left side (column 12). The highest measured well temperatures were found in the wells A-D of column 1 (T_max_ = 36.2-36.9°C), the lowest values in the wells A-F of column 12 (T_min_ = 20.4-20.8°C). The rest of the wells cover the whole temperature range between T_min_ and T_max_. Even though not every MTP row shows exactly the same temperature gradient, a certain repetitive trend can be observed.

By varying the thermostat set temperatures the profile can be shifted to higher or lower temperature ranges. A decrease of T_set,high_ to 40°C results in a temperature range of 17.7-30.3°C, showing that the change of only one set point temperature has influence on both, T_min_ and T_max_ (comp. Figure 3A and B). This is not surprising since the temperature gradient in the thermostating block itself is strongly dependent on the two set point temperatures of the heating and cooling cycle.

The room temperature has a strong influence to the temperature profile, too. Its increase from 30°C to 37°C resulted in a higher temperature range (comp. Figure 3A and C). T_min_ increased from 20.4 to 28.0°C, T_max_ from 36.9 to 39.9°C. This effect is mainly attributed to the MTP’s bottom which is thermally completely exposed to the environment. Hence, there is a large heat exchange area. This cannot be avoided, e.g. by insulation, because the transparent bottom needs to be accessible for the optical measurement. On the other hand, this effect might be beneficial by making the RT an additional temperature control parameter.

Figure 3D and E show further profiles at higher temperature levels. By setting the thermostat temperatures to 5 and 60°C, respectively, well temperatures of 30.3-47.3°C can be realized (Figure 3D). To characterize even thermophilic microbial or enzymatic systems a T_set,high_ of 95°C was chosen (Figure 3E). In this way a profile with T_max_ of 64.5°C was achieved. T_min_ was 42.1°C. It must be considered that under these conditions the cryostat was not able anymore to ensure a set point temperature of 5°C since the cooling water heated up to much while passing through the thermostating block. To work under defined conditions T_set,low_ was increased to 10°C.

In all determined profiles it is obvious that T_min_ and T_max_ differ strongly from T_set,low_ and T_set,high_, respectively. This is due to heat losses in the temperature control system. Some heat transfer might occur from the tubes of the circulation system. Measurements show that this loss is rather low, e.g. for the profile in Figure 3C with T_set,low_ of 5°C and T_set,high_ of 50° the temperatures before entering the block were 5.2°C and 49.3°C, respectively. This proves that the tube insulation worked properly. On the contrary, the current version of the thermostating block has no special insulation to the environment. It can be assumed that the exposed aluminum surface is mainly responsible for the observed temperature differences. The non-insulated MTP bottom is responsible for additional heat dissipation as discussed above. The results in Figure 3F support this argument. Compared to the profile in Figure 3D the MTP was not shaken during the measurement. Interestingly, the differences between the set point temperatures T_set,low_ and T_set,high_ and the regarding minimal and maximal temperatures T_min_ and T_max_ are smaller. This results in a broader well temperature range of 22.2-51.3°C (comp. Figure 3D, 30.3-47.3°C). The phenomenon can be explained by a ventilation effect caused by shaking of the MTP. While an insulating air layer may be generated below a non-shaken MTP decreasing the environment’s influence, this layer may be lost when the plate starts moving. Nonetheless, for most processes sufficient shaking is absolutely necessary due to mixing and mass transfer requirements.For all profiles shown in Figure 3 a slight temperature gradient over the MTP rows (A-H) was observed apart from the intended one over the MTP columns. This is not surprising since the heating water enters the thermostating block closest to well A1, such as the cooling water enters closest to well A12. While passing through the block the heating water gets colder and the cooling water warms up. For the profile in Figure 3C the heating water’s temperature decreased by 1.2 K from entry to exit, whereas the cooling water got 0.5 K warmer. Of course these values will differ from one profile to another.

### Temperature dependence of microbial systems

In the following experiments the information from the temperature profiles (Figure 3) was used to investigate several microbial systems in order to find optimal conditions for biomass formation or recombinant protein expression. Microbial growth (via scattered light) and the formation of fluorescent proteins was followed on-line applying the BioLector technique. To ensure that really the temperature is the limiting factor during the cultivation it was necessary to exclude other limitations. Previous studies showed that the applied media allow non-limiting growth [27,28,31]. A critical factor is the oxygen supply. For the applied shaking conditions (200 μL filling volume, 995 rpm shaking frequency, 3 mm shaking diameter) and aeration with pressurized air a maximum oxygen transfer capacity of approx. 0.03 mol L^−1^ h^−1^ was determined (data not shown). This value differs among media depending on their oxygen solubility and diffusion coefficient [32-34]. However, *E. coli* grown in rich medium at 37°C may require maximum oxygen transfer capacities up to 0.1 mol L^−1^ h^−1^ (data not shown). To realize such high transfer rates the aeration was shifted from pressurized air to pure oxygen. In this way, almost the 5-fold maximum oxygen transfer capacity can be achieved and cultivations without oxygen limitation are warranted for all applied media and microorganisms.

**Figure 4:**
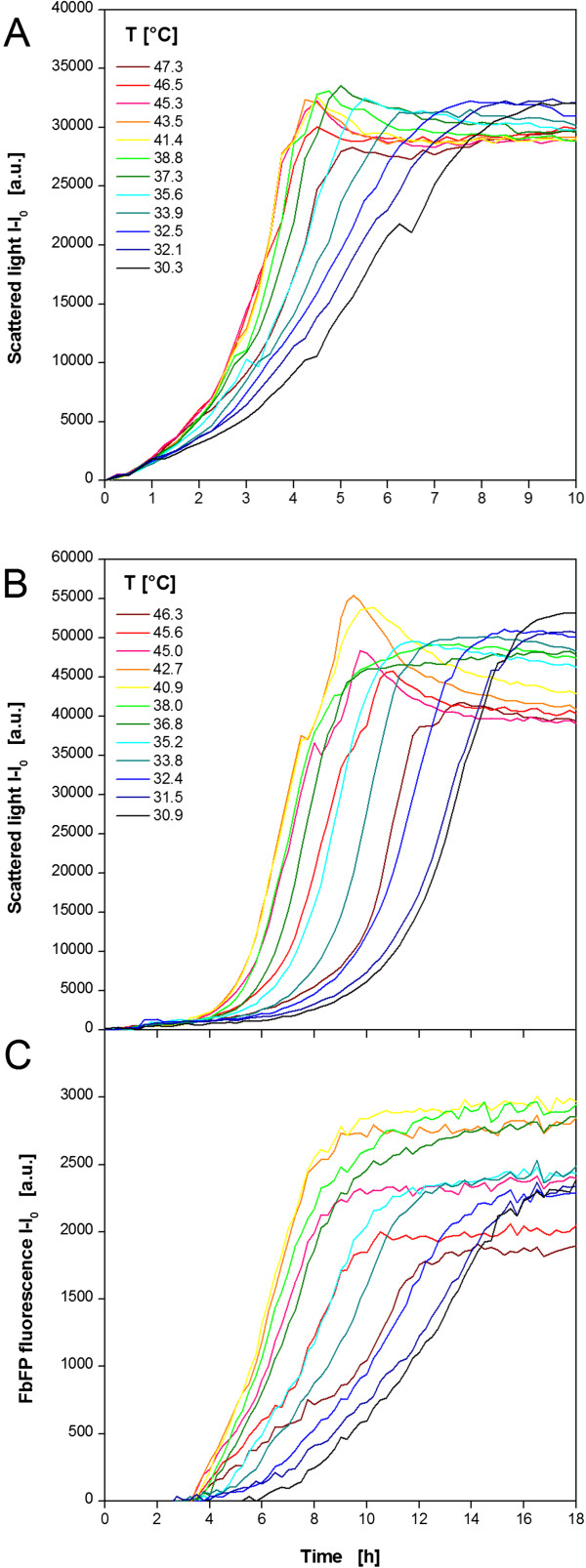
**Cultivation of *E.coli *BL21 expressing the fluorescent protein FbFP applying a temperature profile in a MTP. (A)** Cultivation and online monitoring of microbial growth (via scattered light) in complex TB medium without induction. **(B)** Cultivation and online monitoring of microbial growth and **(C)** fluorescent protein formation in complex auto-induction OnEx. Culture conditions: 96well MTP, V_L_ = 200 μL, n = 995 rpm, d_0_ = 3 mm, aeration with 100% oxygen. Temperature profile: RT = 37°C, T_set,low_ = 5°C, T_set,high_ = 60°C (comp. Figure 3D). Data of 12 (from 48) exemplary wells.

An *E. coli* strain expressing a fluorescent model protein (FbFP) as product was investigated (Figure 4). Non-induced (MTP row A,C,E,G) and induced (MTP row B,D, F, H) cultivations were performed in parallel in one MTP. The temperature profile was essentially identical to Figure 3D. The results of 12 (out of 48) exemplary non-induced cultures in TB medium are shown in Figure 4A. All cultures begin immediately with their exponential growth without any *lag* phase. After 1 h the curves start spreading indicating different growth rates at different temperatures. The lowest rate is observed at the lowest temperature of 30.3°C. Higher temperatures lead to increased growth rates indicated by steeper curves. The maximum growth rate occurs at temperatures of 41.4-45.3°C. A further increase retarded the microbial growth again. Due to varied growth rate the time point for reaching the stationary phase differs as well. At 41.4°C the culture became stationary after 4.5 h, whereas it needed twice as long at 30.3°C. Comparing the final scattered light intensities, it can be seen that slightly more biomass was formed at lower temperatures. This might be explained by a higher energy demand for cell maintenance at higher temperatures which withdraws metabolic resources from growth [35]. It was already found that organisms growing at temperatures above their optimal growth temperature show lower cell yields. Thereby, it was postulated that biosynthetic reactions at high temperatures do not keep pace with catabolic reactions [36].

Exemplary curves for growth and product formation under induced conditions are depicted in Figure 4B and C, respectively. The auto-induction medium OnEx was used which works as follows: glucose is the preferred carbon source and represses recombinant protein expression to ensure undisturbed initial growth. After glucose depletion lactose is taken up and acts as the inducer of the expression system. Glycerol is an additional energy source. The scattered light curves reflect the medium’s working principle (Figure 4B) which was studied before in detail [37]. For all cultures a short *lag* phase (~0.5 h) is followed by a small exponential increase in the scattered light signal after 1.5 h. In this time glucose is consumed allowing undisturbed growth without product formation. As observed for the non-induced cultivations (Figure 4A) the temperature has no influence on the initial behavior of the cells. After the first exponential growth phase the scattered light signal remains at a constant level indicating no further growth. The reason for this is the induction by lactose after glucose depletion. The recombinant protein expression causes a metabolic burden to the cells and inhibits the microbial growth. After a certain time the biomass signal starts increasing again resulting in a second exponential growth phase. From earlier studies it is known that *E. coli* cultures can recover from the metabolic burden after lactose depletion [37]. The duration of the inhibition phase differs over the curves. It is shortest at 40.9-42.7°C and extends at higher and lower temperatures. This might be explained with an accelerated lactose consumption at the temperatures which are most beneficial for the host organism. Consequently, the cells can recover earlier and grow further on the residual glycerol in the medium. For temperatures higher than 38°C the scattered light curves show an unexpected decrease after their maximum value. The reason for this is not yet clear. Morphological changes of the cells when entering the stationary phase might be one explanation [38]. Another could be related to inclusion body formation. It is known that higher temperatures promote protein precipitation in cells [39]. Additionally, there are reports about the influence of inclusion bodies to the cell’s light scatter [40,41]. Further flow cytometric investigations should be performed to finally clarify this issue. The corresponding product formation was followed by fluorescence measurement (Figure 4C). After 3 h all cultures started producing the fluorescent protein FbFP and, subsequently, showed a continuous increase of the fluorescence signal. The curve’s slope depends on the temperature with the strongest increase at 40.9-42.7°C indicating the highest production rate. As a consequence, the maximum FbFP fluorescence is reached in the shortest time after 9 h. The slowest culture is found at 30.9°C reaching its maximum fluorescence after 17–18 h. These times correlate very well with the biomass signal (Figure 4B) which indicates that product formation stops with entering the stationary phase. Additionally, not only the production rate differs among the temperatures, but the maximum product concentration, too. The highest level is observed again at 40.9°C revealing the optimal temperature for FbFP production combining the maximum production rate and product concentration.

**Figure 5:**
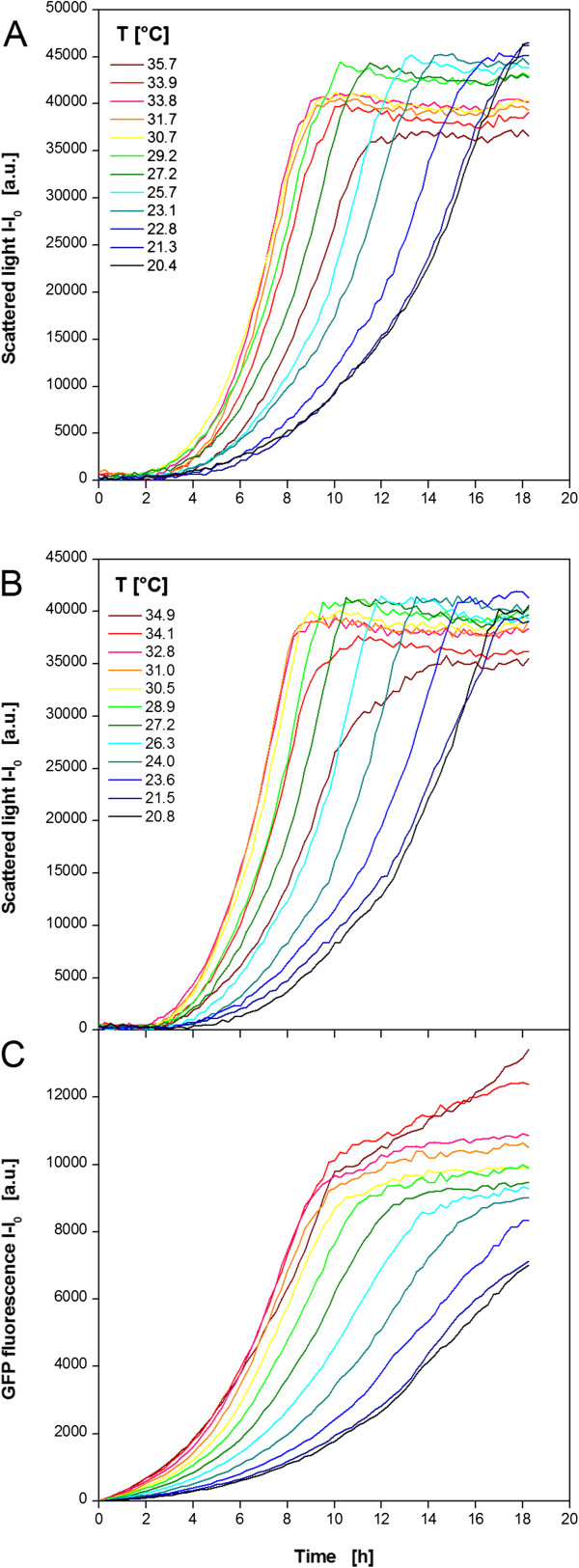
**Cultivation of *K. lactis *GG799 expressing the fluorescent protein GFP applying a temperature profile in a MTP. (A)** Cultivation and online monitoring of microbial growth (via scattered light) without induction in complex YP medium containing 20 g/L glucose. **(B)** Cultivation and online monitoring of microbial growth and **(C)** fluorescent protein formation in complex YP medium containing 20 g/L galactose as substrate and inducer. Culture conditions: 96well MTP, V_L_ = 200 μL, n = 995 rpm, d_0_ = 3 mm, aeration with 100% oxygen. Temperature profile: RT = 30°C, T_set,low_ = 5°C, T_set,high_ = 50°C (comp. Figure 3A). Data of 12 (from 48) exemplary wells.

As a further microbial system the yeast *K. lactis* expressing GFP as product was investigated (Figure 5). Again, non-induced (MTP row A, C, E, G) and induced (MTP row B, D, F, H) cultivations were performed in parallel in one MTP. The temperature profile was essentially identical to Figure 3A. The results of 12 exemplary non-induced cultures in YP medium containing glucose as carbon source are shown in Figure 5A. Contrary to *E. coli*, the cultures show a temperature dependent *lag* phase. The minimum *lag* time of 2 h was observed at 27.2-33.8°C. During the subsequent exponential growth phase also the highest growth rates occur at those temperatures. Shifts to higher or lower temperatures lead to extended lag phases and reduced growth rates. As already observed for *E. coli*, the time point for reaching the stationary phase differs from 9–18 h due to the temperature dependent *lag* phase and growth rate. Again, the final scattered light intensities are higher at lower temperatures as already discussed for *E. coli*.

Contrary to *E. coli*, the growth behavior of *K. lactis* under induced conditions is not much different from that under non-induced conditions (comp. Figure 5A and B). The substitution of glucose by galactose as carbon source and inducer affects the microbial growth insignificantly. The *lag* phase, the growth rate, and the final biomass concentration show the same temperature dependent behavior as observed for the non-induced *K. lactis* cultures. The product formation shows a slightly different trend (Figure 5C). All GFP fluorescence signals increase continuously from the beginning. The strongest increase indicating the highest production rate is observed at 31.0-34.9°C meaning that the GFP expression rather than microbial growth favors higher temperatures. Also the final product concentration increases with rising temperature. The reason for the further increase of the GFP fluorescence in stationary phase observed for 34.1 and 34.9°C is yet unclear. A concentration effect due to increased evaporation cannot explain this phenomenon. The determination of the filling volume after the cultivation revealed relatively low volume decreases of 1.1, 4.5, and 7% at 20.8, 31.0, and 34.9°C, respectively. For this reason, evaporation was not taken into consideration. Higher temperatures may provoke cell lysis in the stationary phase. As a result free GFP may induce brighter fluorescence in the medium without the barrier of the yeast’s cell wall and membrane. Further studies, e.g. by applying flow cytometry, could help to clarify this effect.

For cultivation media it is known that temperature changes may result in pH changes as well, hence, two process parameters are unintendedly varied at once. This might lead to distorted results. The buffers used within this work show rather low changes within the applied temperature profiles with a maximum pH change of 0.2 (0.013 K^−1^). But for more sensitive systems this effect should be considered by adapting the initial pH to the regarding temperature.

**Figure 6:**
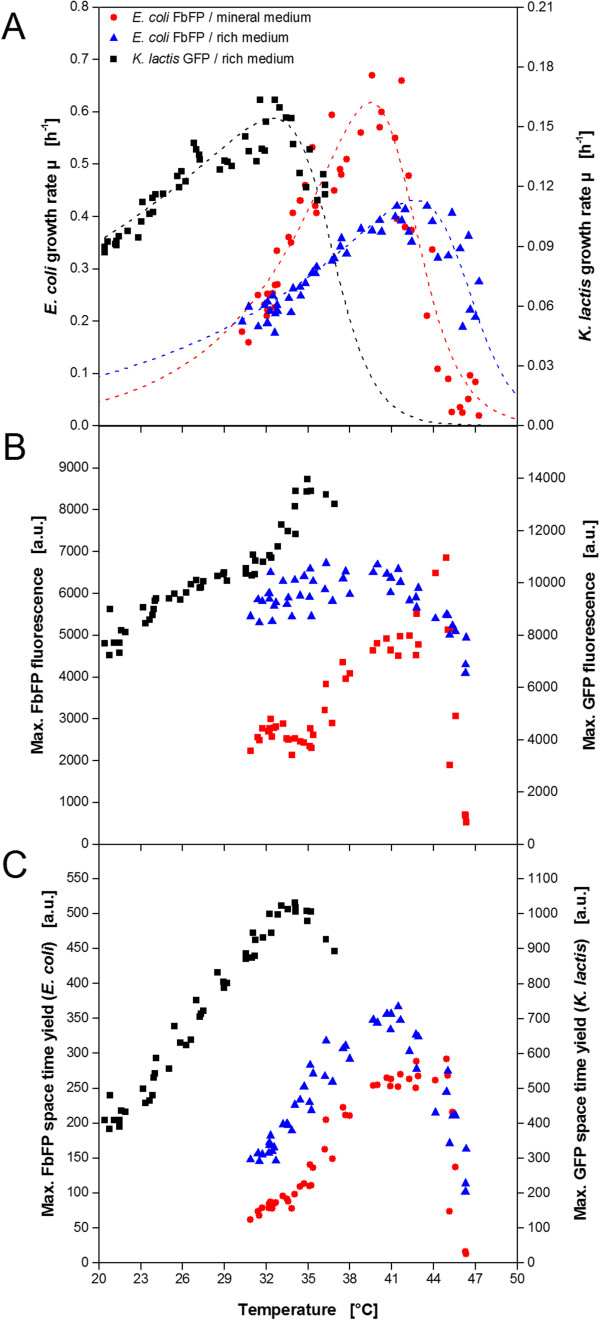
**Determination of the optimal temperature for microbial growth and product formation of *E.coli *and *K. lactis *resulting from temperature profile experiments in MTPs. (A)** Temperature dependent growth rate of *E.coli* (in rich and mineral medium) and *K. lactis* (in rich medium) under non-induced conditions. Temperature dependent maximum product formation **(B)** and maximum STY **(C)** of *E.coli* producing FbFP (in rich and mineral medium) and *K. lactis* producing GFP (in rich medium) under induced conditions. Dotted lines in A indicate Arrhenius fits due to Eq. 1.

From the before described data sets (Figures 4 and 5) it is already possible to get an idea of the temperature optima for microbial growth and product formation in yeast and bacterial cells. But the high throughput of the MTP yields sufficient data for a more detailed characterization. For biomass formation the maximum growth rate under non-induced conditions was chosen as temperature dependent parameter (Figure 6A). As expected, the bacterial and the yeast system show a different behavior. *E. coli* in rich TB medium has a maximum growth rate of 0.41 h^−1^ at 43°C. The highest growth rate determined for *K. lactis* in rich YP medium is 0.16 h^−1^ at 32-33°C. In addition, the growth of *E. coli* in the mineral Wilms-MOPS medium was investigated. Compared to the growth in rich TB medium, a higher maximum growth rate of 0.67 h^−1^ was determined but at a lower temperature of 39.5°C. The observed values are in good agreement with earlier studies about *E. coli*[42-45] and *Kluyveromyces* strains [35,46,47]. For the product formation the maximum product fluorescence (indicator for product concentration) and the maximum space time yield were exemplary chosen as characteristic values (Figure 6B and C). Again differences between the microbial systems and cultivation media occur. *K. lactis* has the maximum product fluorescence and highest STY in the same range of 34-35°C which is slightly higher than the temperature for optimal growth. *E. coli* expressing FbFP in the rich auto-induction medium OnEx shows constant high fluorescence values over a relatively broad range from 35-42°C before it drops sharply at higher temperatures. The regarding maximum STY was determined at 41°C. For FbFP production in the mineral auto-induction medium the highest values for fluorescence and STY are observed at 44°C and 40-45°C, respectively.

An overview of all determined temperature optima is given in Table 2. The optima for growth and product formation are not in agreement for some of the investigated expression systems. Interestingly, even the medium composition has an influence. Compared to the standard cultivation temperatures of 30 and 37°C for *K. lactis* and *E. coli*, respectively, the determined optima differ to some extent. Consequently, an individual temperature screening is recommended guided by the desired aim of the process.It is noticed that the measuring points in Figure 6 show some scattering indicating certain measurement inaccuracies. The fluorescence thermometry method has a standard deviation of ±0.76 K (see chapter Temperature profiles) which might be one explanation. Furthermore, it should be considered that the temperature profiles were determined in separate experiments without the microbial reaction system. It was assumed that no significant changes are encountered during cultivations, thereby, neglecting the possibility of produced reaction heat. In future studies, it should be investigated how fluorescence thermometry can be used directly in the reaction system in order to realize temperature real time monitoring. Nonetheless, the high number of measuring points compensates these disadvantages and gives a clear trend.

**Table 2:** **Temperature optima for growth and recombinant protein expression of *E. coli *(in rich and mineral medium) and *K. lactis *(in rich medium) under non-induced (ni) and auto-induced (ai) conditions**

	**Temperature optimum**		
**Expression system**	**Growth**	**Product concentration**	**Product STY**
*E. coli* (rich medium)	42°C^ni^, 43°C^ai^	35-42°C^ai^	41°C^ai^
*E. coli* (mineral medium)	40.5°C^ni^, 41.5°C^ai^	43-44°C^ai^	40-45°C^ai^
*K. lactis* (rich medium)	32°C^ni,ai^	35°C^ai^	34°C^ai^

**Figure 7:**
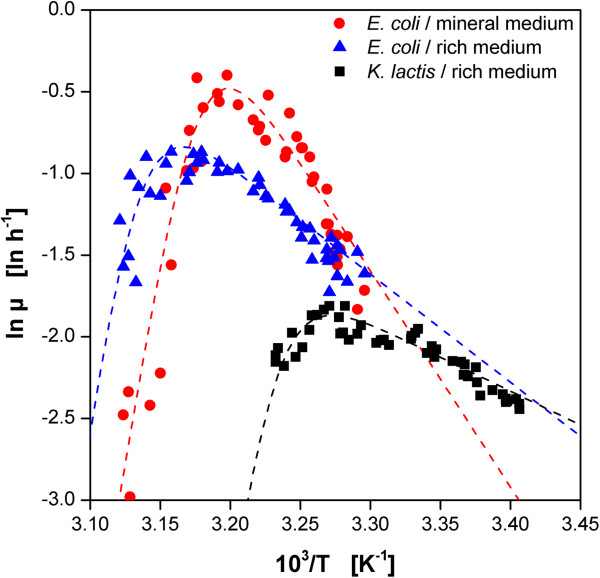
**Characterization of the temperature dependent growth behavior of *E.coli *and *K. lactis *applying the Arrhenius plot.** Temperature dependent growth rates of *E.coli* (in rich and mineral medium) and *K. lactis* (in rich medium) under non-induced conditions (comp. Figure 6A). Dotted lines indicate Arrhenius fits according to Eq. 1.

A typical procedure to describe the temperature dependent behavior of microorganisms and (bio-)catalysts is the Arrhenius plot. An extended version of the Arrhenius equation was used for mathematical modeling (Eq. 1). Thereby, the numerator is the classical Arrhenius equation with the numerical constant A and the activation energy E_g_ describing the typical increase of the growth rate or catalyst activity with increasing temperatures. Assuming that proteins are temperature-denatured and inactivated by a reversible chemical reaction with free energy change (ΔG_d_), the nominator describes the behavior beyond the temperature optimum due to the Hougen-Watson expression for catalyst activity [25]. In Figure 7 the data from Figure 6A for the microbial growth of *E. coli* and *K. lactis* was plotted logarithmically over the reciprocal absolute temperature.

(1) $${\;}_{v_{\max}\;=\;\frac{A\;\exp\left(-E_{g}/\mathrm{RT}\right)}{1\;+\;B\;\exp\left(-\Delta G_{d}/\mathrm{RT}\right)}}$$

By solving Eq. 1 in MS Excel, the parameters for the different investigated microbial systems were determined (Table 3). The model fit for *E. coli* in rich TB medium reveals an activation energy of 55 kJ mol^−1^ which is in very good agreement with earlier reports [42-44]. Interestingly, *E. coli* in the mineral Wilms-MOPS medium shows a much higher value compared to that in TB medium. *K. lactis* shows a lower value of 34.5 kJ mol^−1^. Literature values for *Kluyveromyces* strains range from 36.6 to 85.2 kJ mol^−1^[35,46,47]. In contrast to the activation, the deactivation behavior indicated by ΔG_d_ is similar for all organisms and media.

**Table 3:** **Arrhenius model parameters in Eq.** 1 **for the yeast *K. lactis*, the bacterium *E. coli *and the cellulase preparation Celluclast**

**Parameter**		***E. coli***	***E. coli***	***K. lactis***	**Celluclast**
		**(TB medium)**	**(Wilms-MOPS)**	**(YP medium)**	
E_g_	[kJ mol^−1^]	55	110	34.5	29
ΔG_d_	[kJ mol^−1^]	549	548	539	579
A	[h^−1^]	6 · 10^8^	1.85 · 10^18^	1.3 · 10^5^	1.2 · 10^4^
B		7 · 10^89^	9 · 10^90^	9 · 10^90^	8 · 10^90^

**Figure 8:**
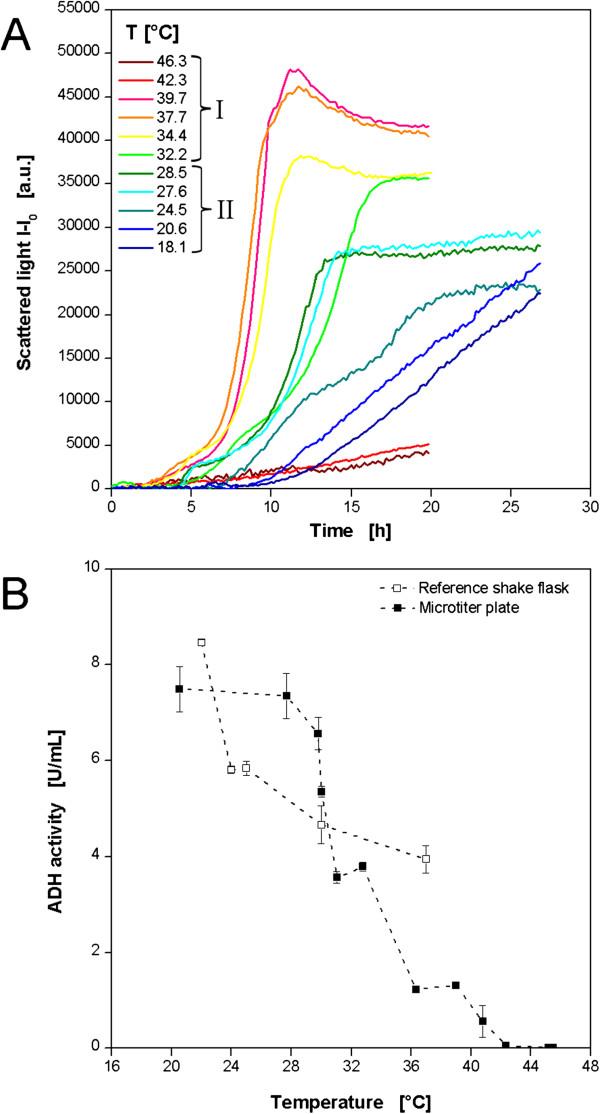
**Cultivations of *E.coli *BL21 expressing the recombinant enzyme ADH-A applying 2 temperature profiles in MTPs. (A)** Cultivation and online monitoring of microbial growth (via scattered light) in complex auto-induction medium OnEx. **(B)** Temperature dependent volumetric activity at the end of the cultivation in the MTP. Reference values from additional cultivations in shake flasks at different temperatures. Culture conditions: 96well MTP, V_L_ = 200 μL, n = 995 rpm, d_0_ = 3 mm, aeration with 100% oxygen. Temperature profile I for T = 32.2-46.3°C: RT = 37°C, T_set,low_ = 5°C, T_set,high_ = 60°C (comp. Figure 3D); Temperature profile II for T = 18.1-28,5°C: RT = 30°C, T_set,low_ = 5°C, T_set,high_ = 40°C (comp. Figure 3B). Reference cultivation: 250 ml shake flask, V_L_ = 10 mL, n = 350 rpm, d_0_ = 50 mm, aeration with air. Online data of 10 exemplary wells.

As an additional expression system *E. coli* producing a recombinant alcohol dehydrogenase A from *Rhodococcus ruber* (ADH-A) was investigated regarding its temperature behavior. In order to look at a broader temperature range, two experimental sets were performed applying a low and a high temperature profile according to Figure 3B and D, respectively. In this way, temperatures of 18.1-46.3°C were realized. To avoid excess evaporation at higher temperatures the cultivation was aborted after 20 h when all cultures had entered the stationary phase. The low temperature profile was applied for 27 h. The biomass formation was followed on-line by scattered light measurement (Figure 8A). As discussed before, the typical growth behavior of *E. coli* in the auto-induction medium OnEx is observed at temperatures of 27.6-39.7°C. After a temperature dependent *lag* phase of 2–4.5 h a first exponential growth is observed. The subsequent growth inhibition indicated by decreasing slopes of the scattered light curves after 4–7 h was caused by the metabolic burden of recombinant ADH-A expression. As seen for *E. coli* expressing FbFP this growth inhibited production phase takes longer at lower temperatures, e.g. 2 h at 39.7°C and 4 h at 27.6°C. When cells recover from that metabolic load a second exponential increase occurs before the stationary phase is reached. At temperatures below 27.6°C the microbial growth becomes more linear so that product formation is not the prior growth inhibitor, but the temperature. At temperatures higher than 39.7°C almost no growth is detected.

Indicator for the product formation was the volumetric ADH-A activity at the end of the cultivation (Figure 8B). An on-line signal was not available and sampling during the cultivation was not possible. Furthermore, the cell suspensions of three wells had to be pooled to have sufficient volume for the enzyme activity assay. Thereby, the information about the respective well temperature was beneficial for combining wells of similar temperature. The results of the MTP experiments show a clear trend of increasing volumetric enzyme activities with decreasing temperatures. Maximum values of 7.4 U mL^−1^ were measured at 20-28°C. At the highest temperatures of 42.3 and 45.5°C no product activity was detected. This correlates very well with the on-line biomass signal where almost no growth occurred in this temperature range (comp. Figure 8A). Taking growth and product formation results together into consideration, a certain trend becomes obvious. Higher temperatures (if not too high) favor bacterial growth but reduce product formation, whereas, at low temperatures the behavior is vice versa. The reference values from the shake flask cultivations do not completely reflect the MTP results. Nevertheless, a similar trend is present with increasing volumetric ADH-A activities at lower temperatures. The maximum activity of 8.5 U mL^−1^ was determined at 22°C.

All these investigations of different microorganisms, expression systems and culture media show how the presented high-throughput temperature profiling helps to find optimal conditions for fermentations. The on-line monitoring feature allows a much more detailed insight to the microbial processes at varied temperature than classical end point analysis, e.g. kinetic parameters such as growth and production rates or space time yields.

### Temperature dependence of cellulases

**Figure 9:**
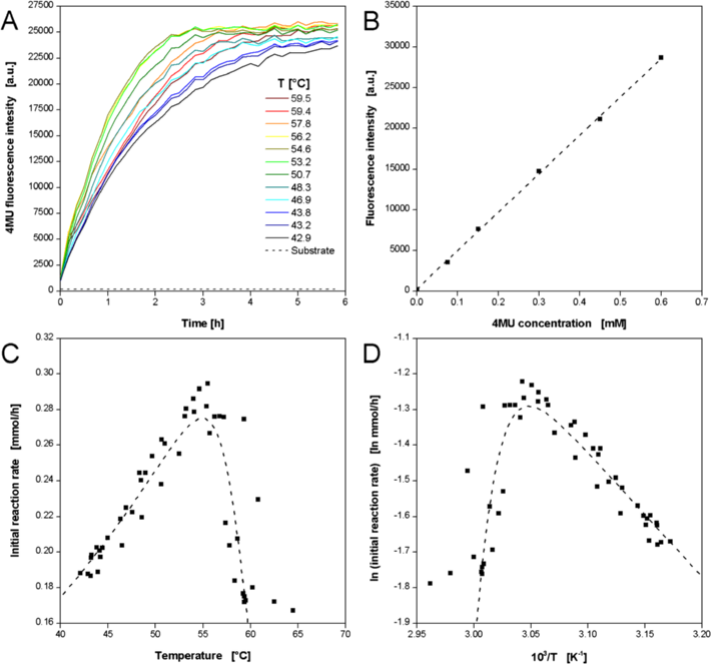
**Enzymatic hydrolysis of 4-methylumbelliferyl-ß-D-cellobioside (4MUC) with cellulase from *T. reseei *(Celluclast) applying a temperature profile in a MTP. (A)** Progresses of 4-methyl-umbelliferyl (4MU) fluorescence (λ_ex_ = 365 nm, λ_em_ = 455 nm) during the hydrolysis of 4MUC at different temperatures. Online data of 12 (from 48) exemplary wells. **(B)** Calibration curve for fluorescence intensity at varied 4MU concentration. **(C)** Temperature dependent initial reaction rate and **(D)** resulting Arrhenius plot of Celluclast. Dotted lines indicate Arrhenius fit. Experimental conditions: 96well MTP, V_L_ = 200 μL, n = 995 rpm, d_0_ = 3 mm, 1 g/L Celluclast and 0.6 mM 4MUC in 0.1 M acetate buffer, pH = 4.8. Temperature profile: RT = 37°C, T_set,low_ = 10°C, T_set,high_ = 95°C (comp. Figure 3E).

As a candidate for the optimization of enzymatic reactions the commercial cellulase cocktail Celluclast was chosen since it is often used for biomass degradation. The Celluclast cocktail contains a mixture of several cellulases from the fungus *Trichoderma reesei*[48]. In order to follow the enzyme reaction on-line, a substrate, namely 4-methylumbelliferyl-β-D-cellobioside (4MUC), was used. It releases the fluorescent dye 4-methylumbelliferone (4MU) when hydrolyzed by cellulases which can be easily detected with a fluorescence spectrometer. Consequently, the 4MUC assay is commonly used for the high-throughput screening of cellulolytic enzymes [49]. Compared to the microbial systems described before, a higher temperature optimum was expected for the cellulases. Consequently, a profile was chosen providing a higher temperature range of 42-65°C (comp. Figure 3E).Twelve exemplary curves resulting from the 4MUC hydrolysis by Cellucalst are depicted in Figure 9A, whereby, the fluorescence intensity indicates the formation of the product 4MU. For all conditions the typical course of enzymatic reactions is observed with a strong increase of the product concentration in the beginning which runs into saturation after a certain time when no substrate is available anymore. The reaction is strongly temperature dependent. The highest reaction rates are observed at 53.2-56.2°C. Consequently, these curves run into saturation already after approx. 3 h. Furthermore, the highest final product concentrations occur at these temperatures. In the temperature ranges above and below reduced reaction rates and extended reaction times for complete substrate conversion are observed. Pure substrate showed a constant signal close to 0 a.u., thereby, proving that no substrate reacted in the absence of enzyme. In order to quantify the cellulolytic reaction, the 4MU fluorescence signal was calibrated assuming that each cleaving of a 4MUC molecule releases one 4MU molecule (Figure 9B). In this way reaction rates as well as final product concentrations can be calculated.

To evaluate the temperature dependent behavior, the initial reaction rate was chosen as indicator and plotted over the respective temperature (Figure 9C). A maximum initial rate of 0.295 mmol h^−1^ was determined at 55°C. At the lowest checked temperature of 42°C an initial reaction rate of 0.19 mmol h^−1^ was found, close to 0.17 mmol h^−1^ at the highest temperature. A complete enzyme deactivation was not achieved. The optimum temperature is in good agreement with other reports about cellulases [3,50,51]. As for microbial systems, the corresponding Arrhenius plot was plotted from these results (Figure 9D). By fitting the curve in MS Excel, the parameters in Eq. 1 for Celluclast were determined (Table 2). It must be considered, that Celluclast is no single cellulase but a mixture of endoglucanases (EG), cellobiohydrolases (CBH) and β-glucosidases (BG). In literature E_g_ values of 20–32 KJ mol^−1^ for EGs [52-54], 18–45 KJ mol^−1^ for CBHs [3,55], and 18–59 KJ mol^−1^ for BGs [55,56] are reported. The here reported value of 29 KJ mol^−1^ is within these ranges.

It can be stated that the presented high-throughput temperature profiling is a useful tool for enzyme characterization. The technique can easily be applied to other enzyme classes as long as fluorescent assays are available. If necessary, the temperature range can be shifted to either higher or lower levels. Sufficient aeration can be assured as discussed for microbial cultivations. With all these presented features the system allows the investigation of a great variety of biocatalysts: psychro-, meso- or thermophilic; oxygen dependent or not.

## Conclusion

Running bioprocesses at their temperature optima is essential for their economical operation. Unfortunately, systems for rapid determination of temperature dependent behavior are rare. The presented high-throughput screening system for temperature optimization faces this challenge. A novel temperature control system with a customized thermostating block and two thermostats can generate individual temperature profiles in MTPs with relatively simple instrumentation. Three controlling parameters were identified: T_set,high_, T_set,low_ and RT. For temperature measurement a fluorescent assay with two Rhodamin dyes was established for the use in MTPs which allows the convenient determination of temperature profiles in short time. Applying the high-throughput screening system BioLector which is already commercially available, important process parameters, e.g. biomass and product formation, are monitored on-line. The high throughput of the MTP provides sufficient data output for a detailed characterization of temperature dependent behavior. Consequently, the combination of the thermostating block, temperature determination via fluorescence thermometry, and an optical on-line monitoring system provides extensive information about temperature dependent process behavior, e.g. concentrations of biomass or products, growth or reaction rates, space time yields, and others. Representative investigations with microbial and enzymatic systems proved the system’s general applicability for various purposes. Thereby, the repetition of the temperature profiles in the MTP rows allows the investigation of several systems in parallel.

During the experiments some potential for improvement became apparent, considering heat dissipation to the surroundings, temperature dependent pH changes, evaporation effects, and sufficient oxygen supply. All these issues can be addressed in future work to achieve most precise results.

The MBR-based high-throughput temperature profiling is a convenient tool for rapid characterization of temperature dependent reaction processes. It allows the fast investigation of numerous conditions, e.g. microorganisms, enzymes, media, and others, in a short time. The simple temperature control combined with a commercial on-line monitoring device makes it a user friendly system.

## Abbreviations

4MU: 4-methylumbellyferone; 4MUC: 4-methylumbellyferyl-cellobioside; A: Numerical constant [h^-1^]; ADH-A: Alcohol dehydrogenase A from *Rhodococcus ruber*; B: Numerical constant [-]; BG: β-glucosidase; CBH: Cellobiohydrolase; E_g_: Activation energy [kJ mol^-1^]; EG: Endoglucanase; FbFP: Flavinmononucleotide-based fluorescent protein; GFP: Green fluorescent protein; I: Measured signal intensity [a.u.]; I_0_: Initial signal intensity [a.u.]; MBR: Micro-bioreactor; MTP: Microtiter plate; OD_600_: Optical density at 600 nm [-]; OnEx: Overnight express Instant TB medium; OTR: Oxygen transfer rate [mol L^-1^ h^-1^]; R: Ideal gas constant ~8.314 [J mol^-1^ K^-1^]; RAMOS: Respiration activity monitoring system; Rh110: Rhodamin 110; RhB: Rhodamin B; RT: Room temperature [°C]; STY: Space time yield [a.u. mL^-1^ h^-1^]; T: Absolute temperature [K]; T_max_: Maximum temperature within a temperature profile [°C]; T_min_: Minimum temperature within a temperature profile [°C]; T_set,high_: Set point temperature of heating thermostat [°C]; T_set,low_: Set point temperature of cooling thermostat [°C]; TB: Terrific broth medium; YP: Yeast peptone medium; ΔG_d_: Deactivation energy change [kJ mol^-1^]; µ: Growth rate [h^-1^]; µ_max_: Maximum growth rate [h^-1^]; v_max_: Maximum reaction rate [h-1].

## Competing interests

The authors declare that they have no competing interests.

## Authors’ contributions

MK made the conceptual design of the study and the experimental setup and methods, performed experiments and prepared the manuscript. CL established the fluorescence thermometry assay in MTPs. SD performed ADH-A expression experiments. WK developed the ADH-A expression system in *E. coli* and provided it for this work. JB assisted with study’s conception, data interpretation and manuscript preparation. All authors read and approved the final manuscript.
